# Gentamicin Released from Porous Scaffolds Fabricated by Stereolithography

**DOI:** 10.1155/2017/9547896

**Published:** 2017-08-20

**Authors:** Somruethai Channasanon, Pareeya Udomkusonsri, Surapol Chantaweroad, Passakorn Tesavibul, Siriporn Tanodekaew

**Affiliations:** ^1^Biomedical Engineering Research Unit, National Metal and Materials Technology Center, Pathum Thani 12120, Thailand; ^2^Department of Pharmacology, Faculty of Veterinary Medicine, Kasetsart University, Bangkok 10900, Thailand

## Abstract

Porous oligolactide-hydroxyapatite composite scaffolds were obtained by stereolithographic fabrication. Gentamicin was then coated on the scaffolds afterwards, to achieve antimicrobial delivery ability to treat bone infection. The scaffolds examined by stereomicroscope, SEM, and *μ*CT-scan showed a well-ordered pore structure with uniform pore distribution and pore interconnectivity. The physical and mechanical properties of the scaffolds were investigated. It was shown that not only porosity but also scaffold structure played a critical role in governing the strength of scaffolds. A good scaffold design could create proper orientation of pores in a way to strengthen the scaffold structure. The drug delivery profile of the porous scaffolds was also analyzed using microbiological assay. The release rates of gentamicin from the scaffolds showed prolonged drug release at the levels higher than the minimum inhibitory concentrations for *S. aureus* and *E. coli* over a 2-week period. It indicated a potential of the scaffolds to serve as local antibiotic delivery to prevent bacterial infection.

## 1. Introduction

Over the last decade, biodegradable materials based on polylactide (PLA) have been extensively studied as scaffolds in tissue engineering. Various techniques have been applied for the fabrication of porous scaffolds suitable for bone tissue engineering including solvent casting, freeze drying, and phase separation [1–7], but they all have suffered limitations in controlling the structure of scaffolds. Porosity and pore size, which are known to play a critical role in bone formation, were hardly reproduced when the scaffolds were fabricated by those techniques. Recently, the fabrication approach using solid free-form technology such as stereolithography (SLA) has demonstrated the advantages of producing scaffolds with controllable porous structure. The computer-controlled solidification of a liquid polymer upon light irradiation, layer-by-layer, has shown the unique ability to precisely fabricate microscaled scaffolds with various architecture and microstructure designs [8–15].

In our previous work, the composite resins of oligolactide and hydroxyapatite (HA) that can be crosslinked by photoinitiated polymerization were developed to obtain scaffolds with designed patterns via SLA fabrication process [16–18]. The fabricated scaffolds appeared to provide appropriate conditions to support the growth of bone cells and their differentiation, making them potentially suitable for bone tissue engineering. The use of these scaffolds as bone implants, however, may encounter a complication from bacterial infection leading to the inflammatory destruction of bone [19, 20] and thus failure in the treatment. Parenteral administration of antibiotics after surgery is unsuccessful in the treatment of bone infections because of the insufficient local penetration of systemic administration. Moreover, the high doses of systemic antibiotics above the minimum inhibitory concentration required at the fracture site cause systemic toxicity [21]. Therefore, imparting the scaffolds a delivery of antibiotics seems offering a better alternative to systemic administration. In this way, high antibiotic concentration is locally delivered to the implanted site. It reduces the time of delivery, avoids fluctuations of the antibiotic concentration through the blood circulation, and eliminates administration of high doses of systemic antibiotics with potential for adverse side effects and systemic toxicity. Although various scaffolds generated using SLA have been reported to support cell proliferation [8, 9, 17, 18, 22], there were only a few reports on investigating these controlled structural scaffolds as carriers for drug delivery.

In this study, the SLA process was applied to fabricate oligolactide/HA scaffolds having the same porosity, but slightly different pore orientation. Gentamicin generally utilized to solve bone infection problems was chosen as the model drug to load into these scaffolds. The porous drug-releasing scaffolds were then investigated for the influences of scaffold structures on mechanical properties as well as drug delivery ability.

## 2. Materials and Methods

### 2.1. Scaffold Preparation

Scaffolds with a dimension of 5 × 5 × 3 mm were fabricated using a stereolithography apparatus equipped with 3W UV laser at 355 nm wavelength and 70 *μ*m laser spot size (model: SLL2020, RP Medical Lab, MTEC Thailand). The photocurable resin used as a scaffold material was (L)-4LM/T 55HA produced in our laboratory [18]. The resin was composed of the 4-arm-methacrylated oligolactide (Mn = 1420 g/mol) and triethylene glycol dimethacrylate (from Esschem Inc.) at the weight ratio of 1 : 1, added with HA (from Taihei Chemical Industrial, Japan) at 55% wt of the resin.

**Figure 1:**
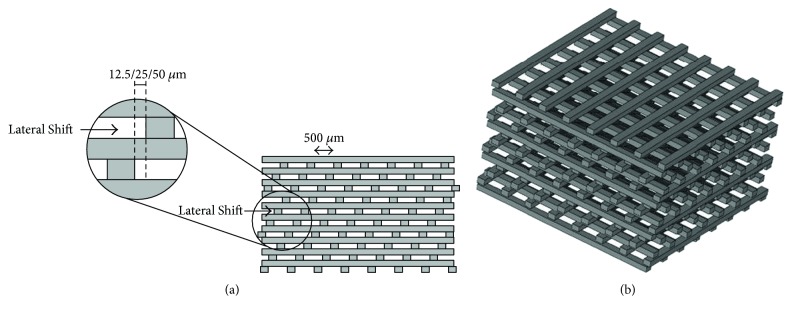
A computer-aided design (CAD) model of the woodpile scaffold: cross-section (a) and 3D structure (b).

Three woodpile scaffolds, lateral shifts of 12.5 *μ*m, 25 *μ*m, and 50 *μ*m, having 71% porosity and 500 *μ*m pore size were designed using a computer-aided design (CAD) as shown in Figure 1. The building parameters were set at a speed of 3.70 mm/sec and a layer thickness of 150 *μ*m. After fabrication, all scaffolds were washed with isopropanol and postcured by irradiating in a UV cabinet for 1 h and boiling in distilled water at 90°C for 1 h. The scaffolds were sterilized with gamma ray at 25 kGy.

The structure of the scaffold was observed under stereomicroscope (ZEISS model Stemi 2000) and field emission scanning electron microscopy (model SU-8030, Hitachi Instruments Inc.).

### 2.2. Porosity Measurement

The porosity of scaffolds was measured by the Archimedes' and image processing methods. For Archimedes' method, each scaffold was weighed dry (W1) using an analytical balance (model GR-200, A&D Co., Ltd) equipped with a pan for making suspended mass measurement. The scaffold was then weighed suspended in distilled water (W2), and this fully water-saturated scaffold was weighed again in air (W3). The porosity of scaffolds was determined as follows:

(1) $$\begin{array}{c} \%\;porosity=\frac{W3-W1}{W3-W2}\times 100. \end{array}$$

For the image processing method, the 3D X-ray images of scaffolds were obtained using a microcomputed tomography (*μ*CT, model: *μ*CT 35, SCANCO Medical AG) with a voxel isotopic resolution of 6.0 *μ*m. The scan was carried out at X-ray energy of 45 kVp and a current of 177 *μ*A. SCANCO *μ*CT software was used to analyze the images and the porosity of scaffolds was calculated as follows:

(2) $$\begin{array}{c} \%\;porosity=\frac{TV-SV}{TV}\times 100, \end{array}$$

where TV = total volume of the scaffold and SV = solid volume of the scaffold.

### 2.3. Compression Test

The scaffolds were compressed using a universal testing machine (model 5943, Instron Corp.) at a crosshead speed of 1.0 mm/min. At least 5 scaffolds were tested and the average compressive strength at break was reported. The compressive strength (CS) of scaffolds was calculated as follows:

(3) $$\begin{array}{c} CS=\frac{F}{A}, \end{array}$$

where *F* = maximum load at break in Newton and *A* = area of specimen on compression in square millimeters.

The slope of the straight-line portion of a stress-strain diagram was recorded as compressive modulus (CM).

### 2.4. *In Vitro* Gentamicin Released from Scaffolds

Gentamicin was loaded by immersing the scaffolds in solution of 40 mg/ml gentamicin sulfate (General Drug House, Thailand) and keeping under vacuum for 10 mins [23]. The scaffolds were then left under a laminar flow hood to completely dry. Drug-loading capacity was calculated as follows:

(4) $$\begin{array}{c} Amount\;of\;drug=\frac{W2-W1}{W2}\times 100, \end{array}$$

where W1 = weight of scaffold and W2 = weight of drug-incorporated scaffold.

An elution study was employed to determine the release characteristics of antibiotics. A phosphate buffer saline (0.1 M PBS, pH 7.4) was used as the dissolution medium. Each scaffold (*n* = 6) was incubated in 1.0 ml of PBS at 37°C for 24 h. The dissolution PBS was collected and 1.0 ml of fresh PBS was added every 24 h for 35 days. All dissolution aliquots were kept at −20°C until analysis. The eluted gentamicin concentrations were characterized by microbiological assay which *Bacillus subtilis* (ATCC 6633) were seeded on antibiotic medium no. 5 (Difco) [24]. Standard gentamicin was diluted with sterile water at concentrations of 0.05, 1, 4, 20, and 40 *μ*g/ml. Each eluted sample was performed in triplicate. The concentration of eluted antibiotic was determined by extrapolation from the standard curve. The lower limit of sensitivity of the assay was 0.05 *μ*g/ml.

### 2.5. Statistical Analysis

Results were expressed as mean ± standard error of the mean. The statistics was calculated using SigmaPlot 11.0. The data were studied by analysis of variance and comparisons between groups were investigated using the Tukey test. Statistical differences were tested at the *P* < 0.05 level.

## 3. Results

### 3.1. Scaffold Fabrication

**Figure 2:**
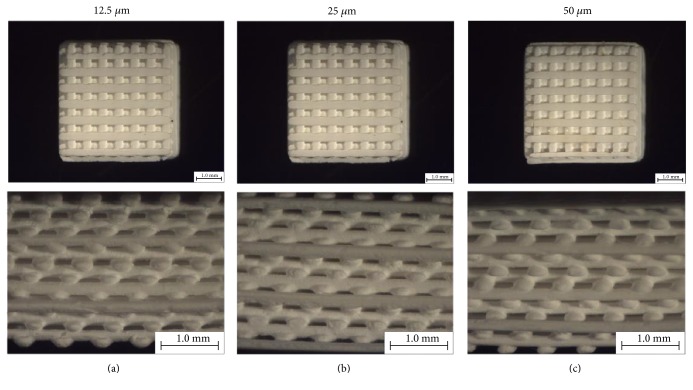
Top view at 10x magnification (top) and side view at 25x magnification (bottom) of the scaffolds observed under stereomicroscope: 12.5 *μ*m scaffold (a), 25 *μ*m scaffold (b), and 50 *μ*m scaffold (c).

**Figure 3:**
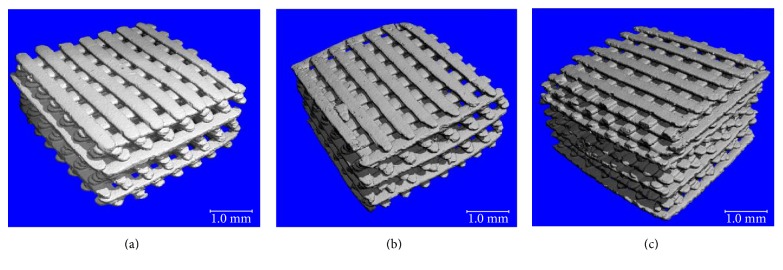
3D images of the scaffolds by microcomputed tomography (*μ*CT): 12.5 *μ*m scaffold (a), 25 *μ*m scaffold (b), and (c) 50 *μ*m scaffold (c).

**Figure 4:**
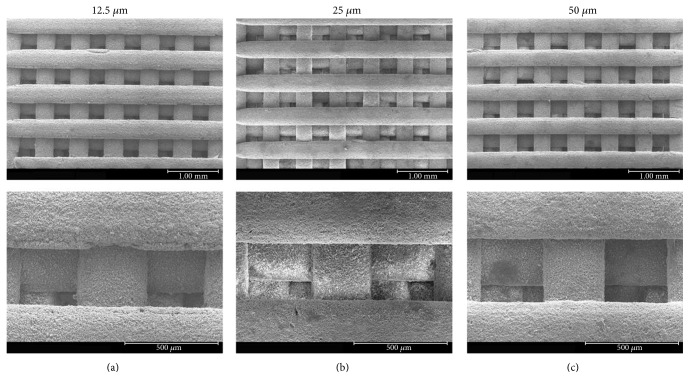
SEM micrographs at 30x magnification (top) and 110x magnification (bottom) of the scaffolds: 12.5 *μ*m scaffold (a), 25 *μ*m scaffold (b), and 50 *μ*m scaffold (c).

**Figure 5:**
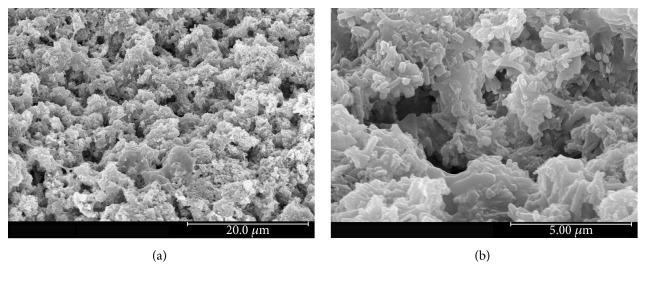
Micropores at the scaffold surface observed by SEM: 2,500x magnification (a) and 10,000x magnification (b).

Images of the built scaffolds under stereomicroscope and their 3D structures constructed by microcomputed tomography (*μ*CT) are illustrated in Figures 2 and 3, respectively. The scaffold structure and pore connectivity which were in good correlation with the corresponding CAD drawings (Figure 1) were observed for each scaffold pattern. A more detailed structure of scaffolds was clearly shown by SEM. As seen in Figure 4, uniform macropores ranging from 300–340 *μ*m with homogeneous arrangement were obtained for each scaffold pattern. In addition to macropores, micropores with pore dimensions in the 1–5 *μ*m size range as well as a homogeneous dispersion of HA rods were also observed in all scaffolds as shown for example in Figure 5.

### 3.2. Physical Characterization

**Figure 6:**
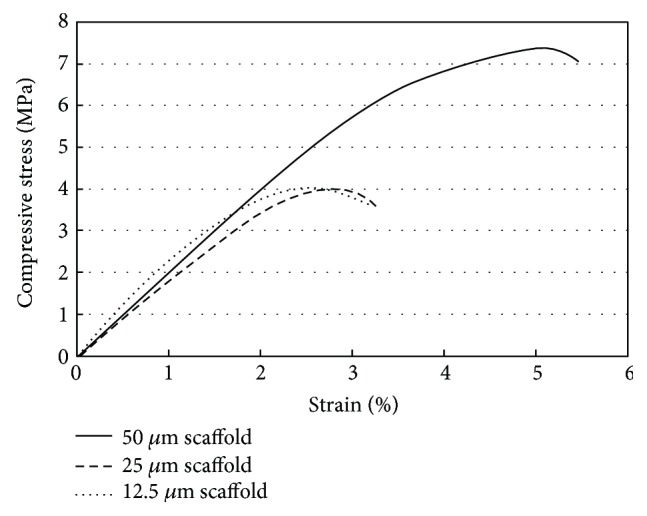
Comparison of mechanical curves of 12.5 *μ*m, 25 *μ*m, and 50 *μ*m scaffolds.

Physical properties of scaffolds, in terms of porosity, compressive strength, compressive modulus, and drug-loading capacity were determined as reported in Table 1. Although with the less percentage, the apparent porosity calculated from Archimedes' principle was in agreement with the porosity determined by *μ*CT in which the slightly higher porosity was obtained as the lateral shift increased. The statistical analysis presented significantly higher porosity of the scaffold with 50 *μ*m shift than of the 12.5 *μ*m scaffold (*P* < 0.05). The scaffold with higher porosity had the higher drug-loading capacity as expected. The plots of stress-strain compared between 12.5 *μ*m, 25 *μ*m, and 50 *μ*m scaffolds in Figure 6 showed brittle characteristics of the scaffolds. There were no significant differences in compressive modulus among three types of scaffolds. Interestingly, the significantly highest compressive strength (*P* < 0.05) was observed for the 50 *μ*m scaffold, the one with the highest porosity.

### 3.3. Drug Release

**Figure 7:**
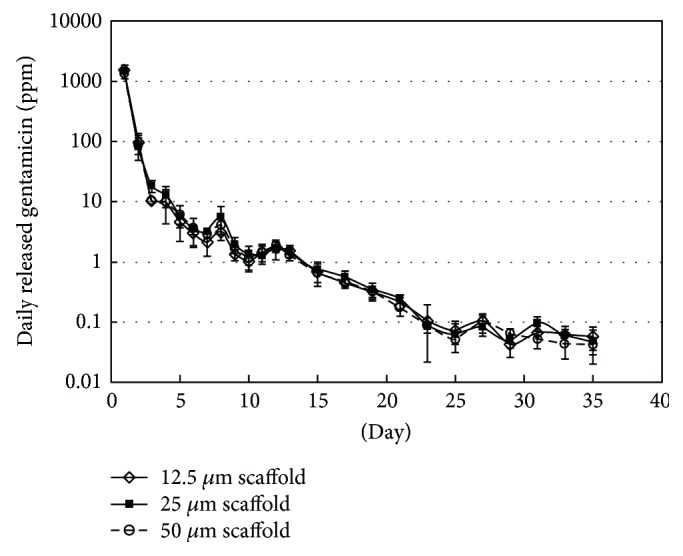
Daily released gentamicin from various scaffolds incubated in PBS at 37°C: 12.5 *μ*m scaffold (◊), 25 *μ*m scaffold (■), and 50 *μ*m scaffold (○).

The daily released gentamicin from the gentamicin-impregnated scaffolds determined by microbiological assay is shown in Figure 7. The microbiological result demonstrated that the drug was still active after being coated on the scaffolds. It was seen that the release of gentamicin followed a typical drug release profile, an initial burst release in a few days followed by a slow release over the next 3 weeks before reaching equilibrium. The released gentamicin from all scaffolds on days 1 and 2 was significantly higher than the other days (*P* < 0.05). However, the released gentamicin from those 3 scaffolds did not differ significantly (*P* > 0.05).

## 4. Discussion

The design and fabrication of scaffolds with a highly porous structure and sufficient mechanical properties is one of the most important issues in bone tissue engineering. Furthermore, loading such antibiotics as gentamicin into the scaffolds would benefit in a local drug delivery to prevent bacterial infection after implant surgery. In this study, woodpile scaffolds with highly interconnected pores were designed and fabricated by the SLA technique. The oligolactide/HA composites which have been previously reported on their biocompatibility [16–18] were used as the scaffold material. All scaffolds, according to the CAD models, had a pore size of 500 *μ*m and 71% porosity with a slight difference in the orientation of pores. The pores were designed not to be stacked up vertically as a straight channel but to shift laterally with respect to each built layer to promote cell entrapment when seeding cells into the scaffolds. Images of the built scaffolds as observed from stereomicroscope, SEM, and *μ*CT showed conformity to the CAD drawings, in terms of uniform pore size and pore interconnectivity, indicating the powerful technique of SLA in fabricating the well-defined scaffold structure. However, the dimensions of the pore did not match the CAD drawings. This was mainly due to the loss of resolution in the SLA fabrication process by light scattering of HA particles. As a result, the scaffolds appeared to be smaller in pore size (300–340 *μ*m) than the design drawings, as evidenced from SEM. This mismatch between the CAD model and the built scaffold could be corrected by including the material-scattering factor together with other scale factors affecting the dimensional accuracy in the CAD software before building the scaffold. The obtained 300–340 *μ*m pore size was, however, in the size range that could provide sufficient space to accommodate cells to proliferate and differentiate to mature bone cells [22, 25, 26]. Moreover, the micropore of 1–5 *μ*m also improved bone growth into scaffolds by increasing the surface area for osteoblast attachment.

With the smaller pore size, the overall porosity of the as-built scaffold was expected to be lower than 71%, the calculated value based on 500 *μ*m pore size. Two techniques, Archimedes' method and *μ*CT imaging, were used to assess the scaffold porosity. Both showed porosity results less than 71% as expected, and the apparent porosity derived from Archimedes' principle was found lower than the calculated porosity by *μ*CT. The porosity obtained by *μ*CT was probably more accurate. This technique constructed the scaffold image from its corresponding cross-sectional segments. Thus, the inner structure of scaffold including pore size and pore interconnectivity was fully assessed, which allowed more accurate determination of porosity. Furthermore, the hydrophobic nature of lactide may cause air to be trapped inside the scaffold resulting in some errors when applying Archimedes' principle. The porosity results ranging from 32 to 38% given by *μ*CT were quite close to the overall porosity of 40% as calculated from the CAD model based on 300 *μ*m pore size. In spite of having such low porosity, there was hardly any problem associated with nutrient and waste flow to facilitate the growth of bone cells in the scaffold since all pores were entirely connected.

Mechanical strength usually inversely relates to porosity [27]. In this study, it was observed differently in the relation between mechanical strength and porosity. The compressive strength was found increased with increasing porosity indicating that not only porosity but also scaffold structure played a critical role in governing the strength of scaffolds. A good scaffold design could allow scaffold to have high porous and high compressive strength. Among all the scaffolds, the 50 *μ*m-shifted scaffold represented the most optimum structure. It yielded compressive strength of 9.24 ± 1.91 MPa which was higher than that of previously reported poly (lactide-co-glycolide) bone and cartilage scaffolds [28]. The high mechanical strength indicated that the structure of the 50 *μ*m scaffold was proper in facilitating load transfer within the scaffold. This was evidenced in the stress-strain curve (Figure 6) that showed ability of the 50 *μ*m scaffold to absorb energy and largely deform prior to fracture. The results also implied that altering the structure or the orientation of pores within the scaffold could be an alternative way to improve the strength of the scaffolds.

Usually, the scaffold with high porosity is expected in facilitating entry of release medium, thereby enhancing drug release. In this study, the released gentamicin from different scaffolds was found similar, although slightly increased porosity was observed in the scaffold with larger lateral shift. Since gentamicin was adsorbed on the scaffold thoroughly, even inside the scaffold by vacuum-assisted method, it implied that the differences in porosity among three scaffold patterns were probably not much to exert an effect on the release of drug. Further investigation of the effect of porosity on drug release may require scaffolds with largely different porosity or structure from one another.

Nevertheless, all scaffolds showed a potential in prolonged drug release over a 3-week period. The hydrophobic nature of lactide-based scaffolds impeded the access of aqueous medium to the inner scaffold resulting in drug retaining for a long-term release. The initial burst release arisen from drug located in the outer surface of scaffold would be satisfactory in killing bacteria, and a continuing release of a lower amount of drug located inside the scaffold would be beneficial in inhibiting bacterial growth. The concentrations of released gentamicin of all scaffolds were higher than the minimum inhibitory concentrations (MICs) for *S. aureus* (ATCC 29213) on day 1–15 and *E. coli* (ATCC 25922) on day 1–13 in which MICs were 0.5 and 1 *μ*g/ml, respectively [29]. As a result, the drug-loaded scaffolds prepared in this study could serve as bone scaffolds with ability to deliver antibiotics to eliminate and prevent bacterial infections for a certain prolonged period.

## 5. Conclusions

The oligolactide/HA scaffolds were fabricated using the SLA process to obtain highly ordered porous structure with uniform pore distribution. The design of the scaffold structure was crucial in obtaining the porous scaffold with high mechanical properties associated to bone scaffolding application. All scaffolds showed a potential in long-term gentamicin release. Therefore, these scaffolds could serve as carriers for local antibiotics delivery to prevent bacterial infection.

## Figures and Tables

**Table 1:** Physical property of scaffolds.

Scaffold	Apparent porosity (%)		CS^∗^ (MPa)	CM^∗^ (MPa)	Drug loading^∗^ (wt %)
	Archimedes^∗^	*μ*CT^∗^			
12.5 *μ*m	19.67 ± 1.52^a^	32.85 ± 1.90^c^	4.93 ± 0.60^e^	40.83 ± 2.60^g^	16.65 ± 4.71^h^
25 *μ*m	24.35 ± 3.85^a,b^	33.71 ± 1.24^c,d^	5.16 ± 1.07^e^	33.58 ± 9.31^g^	22.89 ± 7.15^h,i^
50 *μ*m	24.63 ± 3.23^b^	38.04 ± 1.10^d^	9.24 ± 1.91^f^	35.81 ± 2.73^g^	23.08 ± 4.37^i^

^∗^Values with the same superscript letter are not significantly different (*P* > 0.05).

## References

[1] Chen V. J., Smith L. A., Ma P. X. (2006). Bone regeneration on computer-designed nano-fibrous scaffolds. *Biomaterials*.

[2] Vaquette C., Frochot C., Rahouadj R., Wang X. (2008). An innovative method to obtain porous PLLA scaffolds with highly spherical and interconnected pores. *Journal of Biomedical Materials Research Part B: Applied Biomaterials*.

[3] Grinberg O., Binderman I., Bahar H., Zilberman M. (2010). Highly porous bioresorbable scaffolds with controlled release of bioactive agents for tissue-regeneration applications. *Acta Biomaterialia*.

[4] Rowlands A. S., Lim S. A., Martin D., Cooper-White J. J. (2007). Polyurethane/poly(lactic-co-glycolic) acid composite scaffolds fabricated by thermally induced phase separation. *Biomaterials*.

[5] Montjovent M. O., Mathieu L., Schmoekel H. (2007). Repair of critical size defects in the rat cranium using ceramic-reinforced PLA scaffolds obtained by supercritical gas foaming. *Journal of Biomedical Materials Research, Part A*.

[6] Lee H.-Y., Jin G.-Z., Shin U. S., Kim J.-H., Kim H.-W. (2012). Novel porous scaffolds of poly(lactic acid) produced by phase-separation using room temperature ionic liquid and the assessments of biocompatibility. *Journal of Materials Science: Materials in Medicine*.

[7] Yoon J. J., Kim J. H., Park T. G. (2003). Dexamethasone-releasing biodegradable polymer scaffolds fabricated by a gas-foaming/salt-leaching method. *Biomaterials*.

[8] Melchels F. P. W., Feijen J., Grijpma D. W. (2009). A poly(D,L-lactide) resin for the preparation of tissue engineering scaffolds by stereolithography. *Biomaterials*.

[9] Seck T. M., Melchels F. P. W., Feijen J., Grijpma D. W. (2010). Designed biodegradable hydrogel structures prepared by stereolithography using poly(ethylene glycol)/poly(D,L-lactide)-based resins. *Journal of Controlled Release*.

[10] Melchels F. P. W., Feijen J., Grijpma D. W. (2010). A review on stereolithography and its applications in biomedical engineering. *Biomaterials*.

[11] Jansen J., Melchels F. P. W., Grijpma D. W., Feijen J. (2009). Fumaric acid monoethyl ester-functionalized poly (D,L-lactide)/N-vinyl-2-pyrrolidone resins for the preparation of tissue engineering scaffolds by stereolithography. *Biomacromolecules*.

[12] Channasanon S., Kaewkong P., Uppanan P. (2013). Acrylic-based stereolithographic resins: effect of scaffold architectures on biological response. *Journal of Life Sciences and Technologies*.

[13] Kwon I. K., Matsuda T. (2005). Photo-polymerized microarchitectural constructs prepared by microstereolithography (*μ*SL) using liquid acrylate-end-capped trimethylene carbonate-based prepolymers. *Biomaterials*.

[14] Matsuda T., Mizutani M. (2002). Liquid acrylate-end capped biodegradable poly(e-caprolactone-co-trimethylene carbonate) II. Computer aided stereolithographic microarchitectural surface photoconstructs. *Journal of Biomedical Materials Research*.

[15] Lee S. J., Kang H. W., Park J. K., Rhie J. W., Hahn S. K., Cho D. W. (2008). Application of microstereolithography in the development of three dimensional cartilage regeneration scaffolds. *Biomedical Microdevices*.

[16] Tanodekaew S., Channasanon S., Uppanan P. (2014). Preparation and degradation study of photocurable oligolactide-HA composite: a potential resin for stereolithography application. *Journal of Biomedial Materials Research Part B Applied Biomaterials*.

[17] Tanodekaew S., Channasanon S., Kaewkong P., Uppanan P. (2013). PLA-HA scaffolds: preparation and bioactivity. *Procedia Engineering*.

[18] Channasanon S., Kaewkong P., Uppanan P., Tanodekaew S. (2016). Mechanical and biological properties of photocurable oligolactide-HA composites investigated under accelerated degradation. *Journal of Biomaterial Science, Polymer Edition*.

[19] Lazzarini L., Mader J. T., Calhoun J. H. (2004). Osteomyelitis in long bones. *Journal of Bone and Joint Surgery*.

[20] Mantripragada V. P., Lecka-Czernik B., Ebraheim N. A., Jayasuriya A. C. (2013). An overview of recent advances in designing orthopedic and craniofacial implants. *Journal of Biomedical Materials Research Part A*.

[21] Gitelis S., Brebach G. T. (2002). The treatment of chronic osteomyelitis with a biodegradable antibiotic impregnated implant. *Journal of Orthopaedic Surgery*.

[22] Danilevicius P., Georgiadi L., Pateman C. J., Claeyssens F., Chatzinikolaidou M., Farsari M. (2015). The effect of porosity on cell ingrowth into accurately defined, laser-made, polylactide-based 3D scaffolds. *Applied Surface Science*.

[23] Kawanabe K., Okada Y., Matsusue Y., Iida H., Nakamura T. (1998). Treatment of osteomyelitis with antibiotic-soaked porous glass ceramic. *Journal of Bone and Joint Surgery-British Volume (London)*.

[24] Elsheikh H. A., Osman I. A., Ali B. H. (1997). Comparative pharmacokinetics of ampicillin trihydrate, gentamicin sulphate and oxytetracycline hydrochloride in Nubian goats and desert sheep. *Journal of Veterinary Pharmacology and Therapeutics*.

[25] Gauthier O., Bouler J. M., Aguado E., Pilet P., Daculsi G. (1998). Macroporous biphasic calcium phosphate ceramics: influence of macropore diameter and macroporosity percentage on bone ingrowth. *Biomaterials*.

[26] Bignon A., Chouteau J., Chevalier J. (2003). Effect of micro- and macroporosity of bone substitutes on their mechanical properties and cellular response. *Journal of Materials Science Materials in Medicine*.

[27] Tricoteaux A., Rguiti E., Chicot D. (2011). Influence of porosity on the mechanical properties of microporous-TCP bioceramics by usual and instrumented Vickers microindentation. *Journal of the European Ceramic Society*.

[28] Lee S. J., Lim G. J., Lee J. W., Atala A., Yoo J. J. (2006). In vitro evaluation of a poly(lactide-co-glycolide)–collagen composite scaffold for bone regeneration. *Biomaterials*.

[29] Fluit A. C., Jones M. E., Schmitz F.-J. (2000). Antimicrobial susceptibility and frequency of occurrence of clinical blood isolates in Europe from the SENTRY Antimicrobial Surveillance Program, 1997 and 1998. *Clinical Infectious Diseases*.

